# Role of the Stria Vascularis in the Pathogenesis of Sensorineural Hearing Loss: A Narrative Review

**DOI:** 10.3389/fnins.2021.774585

**Published:** 2021-11-19

**Authors:** Wenting Yu, Shimin Zong, Peiyu Du, Peng Zhou, Hejie Li, Enhao Wang, Hongjun Xiao

**Affiliations:** Department of Otorhinolaryngology, Union Hospital, Tongji Medical College, Huazhong University of Science and Technology, Wuhan, China

**Keywords:** sensorineural hearing loss, stria vascularis, pathogenesis, cell signaling, molecular factors

## Abstract

Sensorineural hearing loss is a common sensory impairment in humans caused by abnormalities in the inner ear. The stria vascularis is regarded as a major cochlear structure that can independently degenerate and influence the degree of hearing loss. This review summarizes the current literature on the role of the stria vascularis in the pathogenesis of sensorineural hearing loss resulting from different etiologies, focusing on both molecular events and signaling pathways, and further attempts to explore the underlying mechanisms at the cellular and molecular biological levels. In addition, the deficiencies and limitations of this field are discussed. With the rapid progress in scientific technology, new opportunities are arising to fully understand the role of the stria vascularis in the pathogenesis of sensorineural hearing loss, which, in the future, will hopefully lead to the prevention, early diagnosis, and improved treatment of sensorineural hearing loss.

## Introduction

Hearing loss is the most common sensory deficit in humans and has a negative influence on 430 million people worldwide, including 34 million children (World Health Organisation, 2021). Most cases of hearing loss are sensorineural and occur due to disease, degeneration, or trauma to the cochlea in the inner ear (Omichi et al., 2019). Common causes of sensorineural hearing loss (SNHL) include hereditary syndromes, non-syndromic hearing loss, presbycusis, and drug-induced hearing loss (Kuhn et al., 2011). The possible pathogeneses include vascular disorders, viral infections, endolymphatic hydrops, and rupture of the labyrinthine membrane (Okada et al., 2013). In addition, the spiral ganglion, organ of Corti, or stria vascularis (SV) are regarded as three major cochlear structures that can independently degenerate and influence the degree of hearing loss (Schuknecht and Gacek, 1993; Ohlemiller, 2004).

**FIGURE 1:**
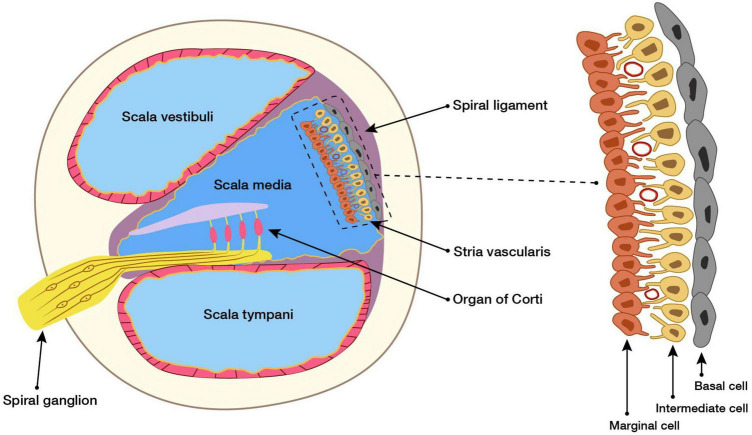
Cross section of the cochlea and structure of SV. Structural and functional damage caused by the SV causes hearing loss, but the mechanism underlying this is unclear. Here, our study reviews the research on the SV in SNHL of different etiologies, and concluded the possible role of the SV in the pathogenesis of SNHL, and aimed to identify new opportunities for the clinical prevention and treatment of SNHL.

The SV is located in the lateral wall of the cochlea, connected externally with the spiral ligament, and internally with the endolymph. The SV is mainly composed of marginal, intermediate, and basal cells (Hibino et al., 2010; Shi, 2016). The structures of the cochlea and SV are shown in Figure 1.

One of the significant functions of the SV is to generate the endocochlear potential (EP), which is essential for audition. It is believed that EP is generated by the transport of potassium from SV into the endolymph (Salt et al., 1987; Wangemann, 1997) in a process involving ion channels and transporters in the SV. Previous studies have found that Na, K-ATPase, Na-K-2Cl-Cotransporter (NKCC), Cl- channels CLCNKA/BSND, and CLCNKB/BSND are located in the basolateral membrane of marginal cells (Lang et al., 2007; Nin et al., 2016), the K + channel KCNQ1/KCNE1 is present in the apical membranes of marginal cells (Hibino et al., 2010), and the inwardly rectifying potassium channels Kir4.1 are expressed in the apical membranes of intermediate cells (Chen and Zhao, 2014). These transporters and ion channels have been shown to be involved in the formation of EP, and inhibition of either transporter or ion channels could reduce EP (Nin et al., 2008).

Another important function of the SV is to regulate the secretion of the endolymph and maintain cochlear homeostasis (Taukulis et al., 2021; Zhang et al., 2021). Among these, H(+) -ATPase (ATP6V1B1 and ATPV0A4) and bicarbonate/chloride transporter SLC26A4 (Pendrin) are expressed in the SV, and regulate the secretion of H+ and HCO3− in the endolymph to maintain pH homeostasis (Mittal et al., 2017); similarly, the Ca(2+) -ATPase and Ca(2+) channels expressed in the SV jointly maintain Ca(2+) homeostasis in the endolymph (Nin et al., 2016).

## Methods

A preliminary literature search up to April 2021 was performed using PubMed, using the retrieval formula “(sensorineur*[Title/Abstract]) AND (hearing[Title/Abstract]) AND (stria vascularis[Title/Abstract])”. The obtained papers were classified by etiology according to the title and abstract. Next, the term “stria vascularis” was combined with the following key-words for further search: hereditary hearing loss, drug-induced hearing loss, age-related hearing loss, noise-induced hearing loss, autoimmune inner ear disease, and systemic disease. Subsequently, irrelevant articles were eliminated and the remaining articles were examined and elaborated on. In addition, the reference lists of the included articles were manually searched for further applicable sources.

## Hereditary Hearing Loss

The SV plays an important role in HHL pathogenesis. Several researchers have previously summarized the histopathologic findings associated with these genetic mutations, and atrophy or fibrosis of the SV has been observed in many cases, often caused by mutations in GJB2, COCH, and MYH9, as well as by Usher syndrome, and Alport syndrome (Bommakanti et al., 2019; Nicolson, 2021). Syndromic and non-syndromic hereditary hearing loss are the two main forms of hereditary hearing loss (HHL) (Egilmez and Kalcioglu, 2016). Table 1 lists the pathological changes and underlying mechanisms of SV in some previously published cases of syndromic hearing loss.

**TABLE 1:** Pathological changes and underlying mechanisms of the SV in different syndromes.

	Relevant background	Pathological changes of SV	Possible underlying mechanisms
Alport syndrome	Alport syndrome is a relatively common heritable basement membrane disorder, caused by mutations in the genes encoding collagen alpha3 (IV), alpha4 (IV), or alpha5 (IV), and usually leads to high-frequency SNHL	Thickening of the capillary basement membrane (Gratton et al., 2005)	• Increase expression of a family of matrix metalloproteinases (MMPs) caused degradation of tight connection in the SV14
			• Pericyte abnormalities (Dufek et al., 2020)
			• Endothelin-1 (ET-1) mediated activation of endothelin A receptors on marginal cells results in thickening of the strial capillary basement membranes (Meehan et al., 2016)
Norrie disease	An X-linked recessive genetic syndrome. The knockout mice showed progressive hearing loss across all frequencies.	Significantly enlarged vessels in the SV, particularly in the apex of the cochlea, and loss of marginal cells. In severe cases, the SV was almost completely degenerated (Rehm et al., 2002)	• Norrin protein activates canonical Wnt signaling by binding to receptor of Frizzled-4 and this signaling system is required for vascular survival in the SV (Ye et al., 2011)
Lysosomal storage diseases	Neuraminidase-1 (Neu1) deficiency is associated with lysosomal storage diseases (LSDs). Hearing loss in Neu1−/− mice involves both conductive and sensorineural components.	Marginal cells of the SV in Neu1−/− cochleae, including invaginations, cavities, and extensive apical vacuolization in marginal cells (Guo et al., 2005; Wu et al., 2010)	
Pendred syndrome	Pendred syndrome is caused by mutations of SLC26A4 and characterized by deafness with enlargement of aqueduct and goiter	SV is significantly enlarged and can be visualized through the pigmentation of the intermediate cells (Xue et al., 2021)	• Upregulation of Bhmt gene caused a disruption of nutrient homeostasis in the endolymph21
			• Reduction of Kcnj10 protein expression under free radical stress in SV (Singh and Wangemann, 2008)
			• Macrophage invasion contributes to tissue degeneration in SV (Jabba et al., 2006)
			• Pendrin dysfunction leads to a loss of KCNJ10 protein expression and a loss of EP (Ahmed jan et al., 2021)
Waardenburg syndrome	Waardenburg syndrome (WS) is an autosomal dominant inherited disease and caused by loss of pigmentary cells in SV of the cochlea, eyes, skin, and hair	Loss of pigmentary cells in SV and absence of EP (Wangemann et al., 2004)	• Mutations in the KIT gene interrupt the development of melanocytes in cochlear, causing SV malformations and dysfunction and ultimately leading to hearing loss (Ni et al., 2013)
			• Lacking of interaction between endothelin 3 and its receptor resulted in abnormal pigmentation and hearing loss in WS4 mice (Matsushima et al., 2002)

Mutations in the potassium channel subunit KCNQ1 cause Jervell and Lange-Nielsen syndrome, while potassium channels in the SV play an important role in maintaining EP (Tranebjaerg et al., 1999). Researchers injected a modified Adeno-associated virus (AAV) construct carrying the Kcnq1 expression box into the endolymph of Kcnq1(−/−) mice, and found that Kcnq1 expression was restored in marginal cells of the SV; EP subsequently returned to normal, and hair cell degeneration was corrected. This was therefore validated as a successful gene therapy treatment for gene defects affecting the function of the SV, which could be of great significance for the future treatment of HHL related to SV dysfunction (Chang et al., 2015).

Most cases of congenital SNHL are non-syndromic. Rohacek et al. (2017) performed whole exome sequencing in individuals with SNHL and identified pathogenic mutations in epithelial splicing regulatory protein 1 (ESRP1). In the cochleae of Esrp1−/− mouse embryos, the cell types in the lateral wall of the cochlear epithelium are altered, resulting in an increase in the number Reissner’s membrane-related cells at the expense of marginal cells. Aberrant splicing of Fgfr2 blocks SV formation because of erroneous ligand usage (Rohacek et al., 2017).

The a4 subunit is a component of the multi-subunit proton pump (H + -ATPase), and mutations in the ATP6V0A4 gene lead to autosomal recessive distal renal tubular acidosis in patients who often show sensorineural hearing impairment. Lorente de Nó et al. studied the inner ear phenotype of Atp6v0a4 knockout mice and observed severe expansion of the scala media, a flattened and thinner SV, and a lack of endocochlear potential, suggestive of defects in SV function. Atp6v0a4−/− mice showed elevated thresholds in auditory brainstem responses. These findings help to understand the role of Atp6v0a4 expression in the ear and may contribute to the development of effective treatments for distal renal tubular acidosis (dRTA)-associated deafness (Lorente-Cánovas et al., 2013).

BDP1 is a member of the TFIIIB complex, which plays a key role in transcription by RNA polymerase III (Girotto et al., 2013). This mitogen-activated protein kinase, MAP3K1, also plays an important role in several cellular processes (Parker et al., 2015), while SCD5 is an endoplasmic reticulum enzyme that plays a crucial role in regulating lipid metabolism (Lu et al., 2020). All of these genes have been reported to be expressed in the SV, while their mutation can cause hearing loss. Further research on the molecular mechanisms underlying deafness associated with these gene mutations is necessary and will provide new opportunities for the diagnosis and treatment of mutation-related hearing loss.

## Drug-Induced Hearing Loss

Currently, more than 150 drugs are known to be ototoxic and can cause functional impairment and/or cellular degeneration of tissues of the inner ear, leading to SNHL (Lanvers-Kaminsky et al., 2017; Guo et al., 2019). The trafficking of ototoxic drugs into the inner ear is generally similar, and all of these drugs must first cross the blood–labyrinth barrier (BLB) of the SV (Salt and Plontke, 2005; Kros and Steyger, 2019); therefore, the SV is considered crucial to their pathogenesis.

Some studies only observed structural abnormalities of SV after treatment with ototoxic drugs, and speculated that the SV was the site of injury to ototoxic drugs. For example, researchers applied drugs such as mitomycin C or taxol to animals, and found degenerative changes in the cochlea, including vacuolization in the SV and a decrease in the number of fibroblasts. Therefore, researchers speculated that subsequent hearing loss in animals is associated with SV (Atas et al., 2006; Moody et al., 2006).

The pathophysiological mechanisms of drug-induced SV dysfunction are poorly understood, although several studies offer some insights. A significant decline in EP is often observed in patients with drug-induced hearing loss. Recently, damage of the Na, K-ATPase, Na-K-2Cl-Cotransporter-1 (NKCC1), and potassium channel KCNQ1, which are related to EP generation, have received attention (Hellier et al., 2002; Xiong et al., 2011). In a mouse model of SNHL induced by co-administration of aminoglycoside and loop diuretics, researchers observed that both the protein and mRNA expressions of α1 and α2 subtypes of Na+ /K+/ATPase and NKCC1 were significantly decreased in the lateral wall, while the expression of KCNQ1 remained unchanged (Salt and Plontke, 2005). These observations provide insight into the detailed mechanisms of EP modulation following SNHL, and may have crucial implications in exploring the mechanisms and future treatment of aminoglycoside-induced SNHL. Recently, the role of inflammation in drug-induced hearing loss has also gained attention. Zhang et al. (2020) found that cisplatin induced the secretion of IL-1 β in SV, suggesting that inflammation is involved in the process of cisplatin-induced SV damage.

At present, the cellular and molecular mechanisms underlying SV injury caused by ototoxic drugs are still unknown, and the pathogenesis of different drugs is not identical. Ion channels in SV are still considered a crucial target for future investigations; inflammation may also be a promising target to prevent and treat drug ototoxicity. However, the detailed mechanisms remain to be investigated.

## Age-Related Hearing Loss

Historical studies have suggested that age-related hearing loss (ARHL) involves a number of auditory structures and mechanisms (Keithley, 2020), including degeneration of the inner and outer hair cells, reduced function of the SV, and degeneration of the auditory nerve (Bowl and Dawson, 2019). ARHL due to functional impairment of the SV is alternatively called strial presbycusis or metabolic presbycusis (Bazard et al., 2021).

One study investigated the morphological changes in the SV of aged mice using TEM, which revealed that the SV in the aged cochlea was degenerated, with a large number of vacuoles and enlarged intercellular spaces (Fetoni et al., 2011; Lyu et al., 2020). However, it is difficult to observe the damage or degenerative changes in the vascular structure of SV using TEM. Carraro et al. (2016) developed a partial corrosion cast method to further investigate the inner ear vasculature, including the capillary structure of the SV and the blood supply of the strial and spiral ligament vessels. Researchers have observed the SV in presbycusis mice using the novel partial corrosion cast technique, and found that the strial vessels in the basal turn were significantly abnormal, being either thin or absent; however, the spiral ligament vessels were normal, which suggests that early pathology started at the level of the SV.

Several mechanisms underlying SV dysfunction in ARHL have been described, including mitochondrial dysfunction, ion transport channel damage, oxidative stress, and inflammation. Lyu et al. observed damaged mitochondria with disorganized dysmorphic cristae and decreased cytochrome C oxidase I (COX1) levels in aged SV, indicating mitochondrial morphological damage and dysfunction (Xiong et al., 2011). Spicer considered that oxidative damage to mitochondria within strial marginal cells causes reduced ATP production, which in turn reduces the Na+ /K+ ATPase activity, leading to reduced EP and elevated auditory thresholds (Spicer and Schulte, 2005).

Because of their important role in generating and maintaining cochlear potential, K+ channels have been repeatedly studied (Peixoto Pinheiro et al., 2021). However, the transport and secretion of K in the inner ear is closely related to Cl-. In a guinea pig aging model, Zhou et al. (2019) found that the expression of mRNA and protein levels of transmembrane protein 16 (TMEM16A), a calcium-activated chloride channel (CaCC), in the cochlear SV decreased, while the auditory brainstem response (ABR) thresholds increased. These findings suggest that the downregulation of TMEM16A may be related to ARHL and provide a potential new direction for clinical prevention and treatment of age-related hearing loss (Zhou et al., 2019). In addition, the results from Kamemori et al. (2002) suggested that the pathogenesis of ARHL may be related to expression of the Klotho protein in SV, which modulates ion transport.

Luz and Jiang conducted experiments in SV-k1 cell lines (from SV) and primary marginal cells of the SV, respectively (Jiang et al., 2018; Rivas-Chacón et al., 2021), to explore the oxidative stress mechanism in SV in ARHL. Recently, researchers have identified age-related changes in the morphology and number of macrophages in the human SV, and suggested that further investigations of the role of macrophage-associated inflammation in SV in ARHL are necessary (Noble et al., 2019).

In the current research, TEM and partial corrosion casting provide a more intuitive understanding of the morphological changes of SV, including some details of vessels in the cochlea. Although there is abundant research on the mechanism of SV dysfunction in ARHL, the exact biological mechanism remains unknown, and thus requires further exploration.

## Noise-Induced Hearing Loss

Noise-induced hearing loss (NIHL) is one of the most common types of SNHL, affecting more than 600 million people worldwide (Le et al., 2017). A previous study demonstrated that exposure to noise could result in a decrease in vessel diameter, an increase in permeability in vessels of the cochlear SV (Goldwyn and Quirk, 1997), and increased macromolecular transport in the SV (Suzuki et al., 2002). However, the underlying mechanism remains unclear.

Abnormal cochlear microcirculation has long been considered a crucial cause of noise-induced hearing loss (Shi, 2011). Normally, healthy cochlear microcirculation supplies blood to the inner ear, removes metabolites, and maintains cochlear homeostasis (Wangemann, 2002). Shin et al. (2019) observed that after exposure to noise, abnormal cochlear microcirculation led to decreased vascular diameter in SV and cochlear ischemia, and increased the expression of catalase, IL-1β, IL-6, and TNF-α in the damaged cochlea. Their findings suggested that cochlear microcirculation is involved in SV damage in NIHL and is associated with oxidative stress and inflammation.

It has previously been shown that NO-derived free radicals in the SV participate in the pathophysiology of NIHL (Chen et al., 2005; Han et al., 2018). Shi et al. explored the apoptotic processes in the SV induced by noise and observed an increase in the production of both nitric oxide and ROS in the SV. Their results provided evidence for a noise–NO–ROS–DNA damage linkage, and indicate that this process leads to marginal cell pathology, inducing blood vessel wall damage and dysfunction of cochlear microcirculation (Shi and Nuttall, 2003).

The role of inflammation in NIHL has also been investigated. Adhesion molecules mediate the adhesion and transmigration of leukocytes, and researchers have found that noise activates the expression of adhesion molecules in the SV to induce hearing loss, leading to speculation that inflammation might be one of the mechanisms by which noise impairs SV function. In addition, Mizushima et al. (2017) observed a significant increase in cells expressing the macrophage-specific protein F4/80 in mouse SV after exposure to noise. They subsequently injected mice with clodronate liposomes to induce apoptosis in macrophages and monocytes, and found that clodronate-treated mice exhibited significantly fewer F4/80-positive cells in the SV and reduced ABR threshold shifts after noise exposure compared to untreated mice. However, IL-1β inhibition did not reverse cochlear damage. These findings suggest a functional link between macrophages and NIHL progression, and indicate that macrophages may be a promising therapeutic target in NIHL (Mizushima et al., 2017).

In addition, there are some other possible mechanisms that could benefit our understanding of the pathology of SV after acoustic trauma, such as abnormal pericytes of SV (Shi, 2009) and regulation of tissue perivascular resident macrophages (PVM/Ms) to BLB integrity (Zhang et al., 2013; Wu et al., 2017).

## Autoimmune Inner Ear Disease

Autoimmune inner ear disease is a rare cause of SNHL (Penêda et al., 2019), and its pathogenesis remains unclear. The SV is generally thought to be an immune site in the inner ear because of the existence of BLB (Das et al., 2019); therefore, many scholars believe that SV injury plays an important role in autoimmune inner ear diseases (Mathews et al., 2003).

Degeneration of SV has been observed in different autoimmune mouse models. Previous studies have reported the destruction of the cochlear BLB and immunoglobulin deposition in SV in C3H/lpr autoimmune mice, resulting in shifts in the ABR threshold (Trune et al., 1989; McMenomey et al., 1992; Wong et al., 1992; Lin and Trune, 1997; Trune, 1997). Similarly, immunoglobulin G deposition in the SV was observed in another autoimmune mouse strain (NZB/kl), and there was a correlation between the degree of hearing loss and the severity of SV lesions (Tago and Yanagita, 1992). Kusakari et al. (1992) have further observed inner ear disorders in MRL/lpr autoimmune mice, including degeneration of intermediate cells, widened intercellular spaces, and immunoglobulin G deposition on the basement membrane of strial blood vessels, as well as in the basal infolding of strial marginal cells. The ABR threshold in MRL/lpr mice was significantly increased (Kusakari et al., 1992). These results suggest that such changes in SV may be the cause of SNHL induced by the immune response.

Ruckenstein et al. conducted a series of studies on MRL-Fas(lpr) mice, which was proposed as a model of autoimmune inner ear disease. They further observed pathological changes in mouse SV, including hydropic degeneration of the SV, antibody deposition in the SV, and reduction in EP, but no reduction in the in Na, K-ATPase levels. Therefore, they inferred that the reduction of EP may occur as a result of cellular degeneration within the SV (Ruckenstein and Hu, 1999; Ruckenstein et al., 1999a,b, c). Furthermore, Trune-treated MRL/LPR autoimmune mice treated with prednisolone or aldosterone showed an improvement in hearing threshold stria and morphology (Trune et al., 2000). These results confirmed that the SV plays an important role in autoimmune inner ear diseases and provide ideas for the clinical treatment of hearing loss induced by the immune response.

Most previous studies observed typical pathological changes in SV, such as edema degeneration and immunoglobulin deposition; however, investigation of the mechanism is lacking and further exploration will be needed in the future.

## Hearing Loss Due to Systemic Disease

The association between type 2 diabetes mellitus and hearing loss remains controversial (Spankovich and Yerraguntla, 2019). SNHL often occurs in patients with type 2 diabetes mellitus (T2DM) (Helzner and Contrera, 2016), and the related cochlear histopathological findings include microangiopathy, and degeneration of the SV and outer hair cells (Fukushima et al., 2006; Samocha-Bonet et al., 2021); however, the mechanism underlying pathogenesis remains unclear (Akinpelu et al., 2014). Tsuda et al. (2016) observed narrowed capillaries and decreased capillary density in SV in Tsumura Suzuki Obese Diabetes (TSOD) mice, which are regarded as a spontaneous type 2 diabetes model. Intracellular edema, dilatation of the intercellular spaces in intermediate cells of the SV, and moderate degenerative changes in the marginal cells of the SV are prominent findings in the cochlea of Zucker diabetic fatty rats. ABR recordings have also shown increased hearing thresholds in diabetic animals (Meyer zum Gottesberge et al., 2015). Mice deficient in the insulin receptor substrate (IRS) develop type 2–like diabetes, while Murillo-Cuesta et al. (2012) observed the traits of SV atrophy, including marginal cell degeneration, dilatation, or merging of the capillaries in IRS2-deficient mice, with profound sensorineural deafness. These results suggest that degeneration of the SV plays a significant role in the pathogenesis of SNHL associated with T2DM.

Congenital and acquired hypothyroidism can result in hearing loss (Psaltakos et al., 2013; Tsai et al., 2020), while in the cochlea of animals with hypothyroidism, large intercellular spaces in the SV with degeneration of marginal and intermediate cells, and inner and outer hair cell degeneration are often observed. Laboratory animals showed increased auditory thresholds. These findings support the idea that SV injury is related to SNHL induced by hypothyroidism (Meyerhoff, 1979).

Several researchers have observed the cochlear structures in mice with hyperlipidemia and found profound edema in SV (Gratton and Wright, 1992; Satar et al., 2001). When viewing the inner ear of hamsters with hyperlipidemia using transmission electron microscopy, many protrusions toward the endolymphatic space on the surface of marginal cells of the SV and vascular degeneration of the marginal cells and intermediate cells of the SV were observed. These results indicate that hypercholesterolemia can lead to functional changes in the cochlea and SNHL (Hidaka, 1997).

Systemic diseases such as diabetes, hypertension, and hyperlipidemia can all cause hearing loss. However, current studies have only proven that the SV is the damaged site of the cochlea, while the underlying pathogenesis is still unknown.

## Hearing Loss Caused by Other Reasons

Cytomegalovirus (CMV) infection is one of the most common causes of congenital hearing loss in children (Prosser et al., 2021) and its pathogenesis is poorly understood (Schleiss, 2011). As mentioned above, Carraro et al. (2017) also used this novel partial corrosion cast technique in mice with CMV infection to explore possible vascular involvement associated with hearing loss (Keithley, 2020). Researchers have observed a significant reduction in vessel diameter in the capillaries of the stria, suggesting that the first affected structures were the strial vessels. Subsequently, Carraro et al. (2017) further investigated mice in which the cerebral cortex was inoculated with CMV and found that after the virus migrated to the inner ear, most damage was found in the SV at the cochlear apex, including vascular degeneration, loss of capillary network, narrowing of the capillary lumen, while more damaged cochleae show further degeneration toward the base. Some mice infected with CMV completely lost the SV of the cochlear apical vessels. Further, significant elevation in the ABR and distortion product otoacoustic emission (DPOAE) thresholds were observed in CMV-treated mice. It has been suggested that initial auditory threshold loss may be related to strial dysfunction (Carraro et al., 2017). In a similar animal model, Li et al. (2014) observed hyperemia in the SV and spiral ligament, as well as bleeding in the scala vestibule and scala tympani. It could thus be speculated that hearing loss in mice after injection with CMV is associated with permeability changes in the BLB (Li et al., 2014). In another mouse model that simulated human CMV infection, CMV-infected mice were observed to have significant atypical hyperplasia in the cochlear striatum, including downregulation of KCNQ1 protein expression in the striatum, and dysplastic and malformed melanocytes (Melnick and Jaskoll, 2013). In addition, Li et al. (2008) observed viral infection in the SV when establishing an experimental model of CMV-induced hearing loss in newborn mice. These findings reveal a link between SV injury and CMV-induced hearing loss. It could thus be inferred that the SV may be the first cochlear structure to be damaged and may play a significant role in the pathogenesis of CMV-induced hearing loss.

Temporary or permanent SNHL is a sequela of pneumococcal otitis media. Tsuprun et al. (2008) inoculated the middle ear of chinchillas with wild-type Streptococcus pneumoniae, and histological analysis revealed no fluid, inflammatory cells, or bacteria in the middle ear, indicating SNHL. Histopathology of the inner ear revealed edema and injury of the SV, while hair cells and the spiral ganglion seemed to be intact, suggesting that the SV may be the target of otitis media injury, leading to SNHL (Tsuprun et al., 2008).

In Mongolian gerbils with *Streptococcus pneumoniae* meningitis, Aminpour et al. (2005) observed the complete destruction of SV and moderate to severe hearing loss, and similar results were observed in a rabbit model of pneumococcal meningitis (Rappaport et al., 1999). Stokroos et al. (1998) further observed SV degeneration and rapid hearing loss in guinea pigs infected with herpes simplex virus type 1 (HSV-1) labyrinthitis. These results suggest that SV impairment is associated with hearing loss secondary to bacterial or viral infection, and may have great significance in the early detection and treatment of hearing loss.

Infection with CMV or *Streptococcus pneumoniae* may lead to SNHL. At present, it could be inferred that SV damage is related to hearing loss. However, the exact mechanism remains to be elucidated.

## Discussion

The morphological changes and functional impairment of the SV are observed in the pathogenesis of SNHL. Some types of hearing loss share commonalities in their pathogenesis. For example, all ototoxic drugs must cross the BLB to enter the inner ear tissues to exert their cytotoxic effects and induce hearing loss; therefore, extensive vacuolization in the SV has always been observed in related investigations. Moreover, the inner ear has been considered an immune-privileged site because of the presence of BLB (Rivas-Chacón et al., 2021). Autoimmune inner ear disease usually manifests as immunoglobulin G deposition in the SV. However, it is difficult to find commonalities in the pathogenesis of other types of hearing loss, due to the high heterogeneity; indeed, 200 deafness-related genes were identified (Lang et al., 2007). Hereditary SNHL is associated with complicated and diverse mechanisms. In addition, ARHL is a multifactorial process, each factor has notably different effects between individuals (He et al., 2019), and only a few factors in this process have been researched. Thus, this research represents only “the tip of the iceberg,” and it is difficult to formulate a network structure.

The cochlea’s small size, fragility, and encasement in the densest bone in the body located deep within the skull make it difficult to dissect and explore (Ren et al., 2019); thus, it has not been thoroughly explored in the current literature. With the development of microscopic imaging techniques and improved molecular biology experimental technology, morphological studies have become increasingly available, and have provided us with a new opportunity to perform research on the function of the stria vascularis, for example, investigating the endocochlear potential and ion channels.

Although the current research on the SV in SNHL is still fragmented and unsystematic, we believe that the SV plays an important role in SNHL and look forward to further investigations on the mechanisms underlying of SNHL, which should identify new targets for the prevention and treatment of SNHL.

## Author Contributions

WY and SZ wrote the manuscript. All authors contributed to the article and approved the submitted version.

## Conflict of Interest

The authors declare that the research was conducted in the absence of any commercial or financial relationships that could be construed as a potential conflict of interest.

## Publisher’s Note

All claims expressed in this article are solely those of the authors and do not necessarily represent those of their affiliated organizations, or those of the publisher, the editors and the reviewers. Any product that may be evaluated in this article, or claim that may be made by its manufacturer, is not guaranteed or endorsed by the publisher.

## Abbreviations

AAV: adeno-associated virus
ABR: auditory brainstem response
ARHL: age-related hearing loss
BLB: blood–labyrinth barrier
CaCC: calcium-activated chloride channel
CMV: cytomegalovirus
COX1: cytochrome C oxidase I
DPOAE: distortion product otoacoustic emission
dRTA: distal renal tubular acidosis
EP: endocochlear potential
ESRP1: epithelial splicing regulatory protein 1
ET-1: endothelin-1
HC: hair cell
HHL: hereditary hearing loss
HSV-1: herpes simplex virus type 1
IRS: insulin receptor substrate
LSDs: lysosomal storage diseases
MPS: mucopolysaccharidosis
Neu1: neuraminidase-1
NIHL: noise-induced hearing loss
NKCC: Na-K-2Cl-Cotransporter
NKCC1: Na-K-2Cl-Cotransporter-1
PVM/Ms: tissue perivascular resident macrophages
SNHL: sensorineural hearing loss
SV: stria vascularis
TEM: transmission electron microscopy
TMEM16A: transmembrane protein 16
TSOD: Tsumura Suzuki Obese Diabetes
T2DM: type 2 diabetes mellitus.
